# Normalization of the tumor microenvironment by harnessing vascular and immune modulation to achieve enhanced cancer therapy

**DOI:** 10.1038/s12276-023-01114-w

**Published:** 2023-11-01

**Authors:** Yechan Choi, Keehoon Jung

**Affiliations:** 1 Department of Biomedical Sciences, Seoul National University College of Medicine, Seoul, 03080 Republic of Korea; 2 Department of Anatomy and Cell Biology, Seoul National University College of Medicine, Seoul, 03080 Republic of Korea; 3 Institute of Allergy and Clinical Immunology, Seoul National University Medical Research Center, Seoul, 03080 Republic of Korea

**Keywords:** Cancer microenvironment, Cancer immunotherapy

## Abstract

Solid tumors are complex entities that actively shape their microenvironment to create a supportive environment for their own growth. Angiogenesis and immune suppression are two key characteristics of this tumor microenvironment. Despite attempts to deplete tumor blood vessels using antiangiogenic drugs, extensive vessel pruning has shown limited efficacy. Instead, a targeted approach involving the judicious use of drugs at specific time points can normalize the function and structure of tumor vessels, leading to improved outcomes when combined with other anticancer therapies. Additionally, normalizing the immune microenvironment by suppressing immunosuppressive cells and activating immunostimulatory cells has shown promise in suppressing tumor growth and improving overall survival. Based on these findings, many studies have been conducted to normalize each component of the tumor microenvironment, leading to the development of a variety of strategies. In this review, we provide an overview of the concepts of vascular and immune normalization and discuss some of the strategies employed to achieve these goals.

## Vascular-immune modulation: a new frontier in cancer therapy

New insights into vascular and immune normalization strategies show potential for improving cancer therapy outcomes. In this review, authors discuss the current state of research on vascular and immune normalization in cancer treatment, highlighting the intricate relationship between these two processes. Vascular normalization aims to restore the integrity of tumor blood vessels, improving oxygenation and drug delivery, while immune normalization focuses on modulating the tumor immune microenvironment to suppress immunosuppression and recruit anti-tumoral immune cells. Various methods for achieving vascular normalization include targeting VEGF signaling, Ang-Tie signaling, oncogenic signaling in cancer cells, and even CD4 + T-cells. Strategies for immune normalization involve modulating the myeloid compartment, enhancing effector T cell function through cancer vaccines or immune checkpoint blockade, and using oncolytic viruses to target tumor cells. Combining these approaches may yield even greater efficacy in cancer treatment.

## Introduction

Cancer is characterized by the uncontrolled growth and spread of cells harboring genetic or epigenetic abnormalities^1^. Viewing cancer solely as a collection of malignant cells fails to capture the intricate processes occurring within tumors. Increasing evidence supports the notion that cancers should be regarded as disorganized “pseudo-organs” engaged in constant interactions with their surrounding milieu^2^. From this, the concept of the tumor microenvironment (TME) has emerged. The TME encompasses not only the tumor cells but also the neighboring cellular and structural components of the tumor, including immune cells, blood vessels, stromal cells such as fibroblasts, and extracellular matrix (ECM)^3,4^. Furthermore, the TME does not exhibit a fixed phenotype, instead varying substantially with tumor type, host factors, and disease progression and showing dynamism even within an individual host. Within the TME, complex interactions often give rise to conditions of hypoxia, low pH, and elevated interstitial fluid pressure (IFP), fostering an immunosuppressive environment that facilitates tumor progression, metastasis, and resistance to anticancer therapies^5–7^. For instance, hypoxia, a hallmark of the TME of various cancer types, can attenuate the efficacy of chemotherapy and radiotherapy^8,9^, trigger the secretion of immune-suppressive cytokines, and promote the recruitment and proliferation of immune-regulatory cell populations^10^. Numerous studies have focused on targeting factors contributing to these effects to augment the effectiveness of anticancer therapies.

Initial attempts to target these factors proved inefficient and, in some instances, even exacerbated tumor progression^11,12^. Notably, the widespread use of antiangiogenic therapy (AAT), initially developed to eliminate tumor blood vessels, often results in excessive vessel pruning, aggravating hypoperfusion and hypoxia, thus enhancing tumor aggressiveness^11^. These observations suggest that, rather than depletion, a strategy of normalization may yield superior outcomes^12–14^. Additionally, there is growing recognition of the crucial role played by the immune microenvironment in tumor progression and therapy response. Strategies aimed at normalizing the tumor immune microenvironment (TIME) have gained much attention as potential therapeutic interventions^15^. These approaches encompass modulation of immune checkpoints, recruitment and activation of effector immune cells, and suppression of immunosuppressive cell populations, with the ultimate goal of enhancing antitumor immune responses and restoring immune surveillance against cancer^15^. In this review, we categorize current normalization approaches into two main groups: vascular normalization and TIME normalization. We discuss the characteristic features of the TME associated with each strategy and provide a concise overview of ongoing research targeting these strategies.

## Vascular normalization

### Background

The rapid growth of solid tumors necessitates an increased supply of oxygen and nutrients. To meet this demand, tumors stimulate the sprouting of blood vessels from preexisting vessels, that is, angiogenesis^2,16–18^. Tumor angiogenesis arises from an imbalance between proangiogenic and antiangiogenic factors, often favoring the former^14,19^. Consequently, the vasculature that forms within tumors is heterogeneous and poorly perfused, comprising abnormal and leaky blood vessels^17,19,20^. Blood flow through this irregular vasculature is stagnant and variable, leading the tumor environment to be characterized by hypoxia, low pH, and elevated IFP^21–23^. Moreover, in certain solid tumors, cancer-associated fibroblasts (CAFs) and their production of ECM components exert compressive forces on tumor vessels, exacerbating the hypoxia^24^. This TME facilitates the recruitment and expansion of immune-suppressive cell populations^25^ and pathological CAFs^26–28^, hampers the cytotoxic activity of tumor-infiltrating effector T cells, enables immune evasion by cancer cells^8,29^, promotes metastasis^30^, and diminishes the efficacy of radiotherapy^31^, chemotherapy^32^, and immunotherapy^33^.

**Fig. 1:**
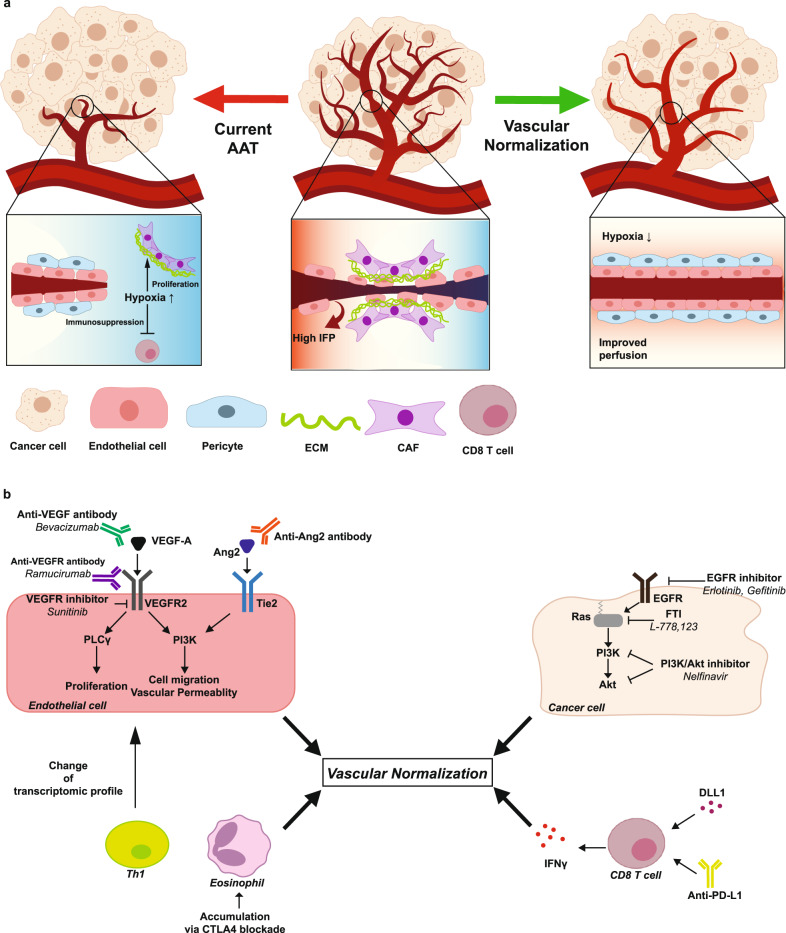
Vascular normalization: Restoring vascular integrity to enhance oxygenation and therapeutic drug delivery in tumor vasculature. **a** Comparison of vascular normalization with current therapies using AATs. Tumors exhibit abnormal vasculature, characterized by irregularly shaped endothelial cells, sparse pericyte coverage, and compression by tumor cells and their extracellular matrices, resulting in poorly perfused vessels (middle panel). Current antiangiogenic therapies, mainly aimed at depleting the tumor vasculature, often lead to excessive vessel pruning, causing hypoxia and limited drug delivery (left panel). Vascular normalization strategies aim to restore vascular integrity, improving oxygenation and enhancing the delivery of therapeutic drugs (right panel). **b** Various vascular normalization methods. Vascular normalization can be induced by targeting VEGF signaling or Ang–Tie signaling in endothelial cells or oncogenic signaling in cancer cells. Immune checkpoint blockade can induce vascular normalization through the function of CD8+ or CD4 + T cells and the accumulation of eosinophils. AAT, antiangiogenic therapy; IFP, interstitial fluid pressure; CAF, cancer-associated fibroblast; ECM, extracellular matrix; VEGF, vascular endothelial growth factor; VEGFR, vascular endothelial growth factor receptor; PLCγ, phospholipase γ; PI3K, phosphoinositide 3-kinase; Ang2, angiopoietin 2; EGFR, epidermal growth factor receptor.

Initially, AATs were developed with the intention of inhibiting tumor growth by disrupting the blood supply and oxygen delivery to tumors^34,35^. However, the use of AATs alone was not successful at combating cancer, exhibiting promising results only when combined with other agents, including chemotherapeutics^36,37^. These unexpected outcomes were termed the “AAT paradox“^21^, and the concept of “vascular normalization” was proposed by Rakesh K. Jain in 2001 to explain this phenomenon^38^. The vascular normalization hypothesis holds that the structure and function of tumor vessels can be restored by employing low doses of AATs within a specific time frame. This restoration process improves oxygen delivery and enhances the penetration of therapeutic drugs^39–41^. Although both conventional AAT-induced vessel pruning and vascular normalization result in overall decreased vessel density, the underlying mechanisms differ substantially^23^. Conventional AAT reduces vessel density through vessel depletion, whereas vascular normalization improves vessel function, manifested by enhanced vessel perfusion, increased pericyte coverage, and reduced hypoxia^23,42,43^ (Fig. 1a).

One of the notable advantages of vascular normalization is its potential for synergizing with other anticancer treatments, including chemotherapy^44,45^, radiotherapy^46^, and immunotherapy^2,47,48^. For instance, a phase II clinical trial in HER2-negative breast cancer patients demonstrated that the combination of bevacizumab (an anti- vascular endothelial growth factor (VEGF)-A antibody) with adriamycin/cyclophosphamide/paclitaxel chemotherapy exhibited vascular normalization effects and was associated with tumor regression^44^. In another phase II clinical trial, bevacizumab showed synergistic effects with lomustine, a chemotherapeutic agent, in patients with recurrent glioblastoma (GBM)^49^.

Achieving vascular normalization poses certain challenges. The optimal dose of AAT and the timing of effective normalization, known as the “normalization window,” are tightly limited and vary between individuals, which presents obstacles to its clinical application^21^. Recent research has unveiled various approaches to induce vascular normalization, ranging from targeting the well-known VEGF signaling pathway to utilizing helper T cells (Fig. 1b). In the next section, we will introduce some of the numerous targets that can be exploited for vascular normalization.

### VEGF signaling remains the major target in vascular normalization

Many solid tumors overexpress factors involved in the VEGF signaling pathway, a key feature in their pathological angiogenesis^50,51^. Targeting the ligands or receptors within this pathway has demonstrated vascular normalization effects in various types of cancer. VEGF-A is one of the best-studied ligands in the context of tumor angiogenesis. Initial attempts to target this VEGF ligand involved the use of A4.6.1, a murine anti-human VEGF IgG. A4.6.1 demonstrated the capability to normalize tumor vasculature in different cancer models, including colorectal cancer, glioblastoma, and melanoma xenografts^52^. Furthermore, this antibody enhanced the effectiveness of chemotherapy and radiation therapy when used in combination with each modality^53^.

Another notable example is bevacizumab (Avastin, Genentech/Roche), a humanized monoclonal anti-VEGF IgG that has received regulatory approval from the US Food and Drug Administration (FDA) for first-line treatment of metastatic colorectal cancer, non-small-cell lung cancer (NSCLC), metastatic renal cell carcinoma, and recurrent GBM in combination with other anticancer therapies^54^. Studies utilizing bevacizumab have demonstrated vascular normalization effects in melanoma, breast carcinoma, ovarian carcinoma, and GBM^39,55,56^. Many studies highlighting the synergistic effects of vascular normalization with other therapies have been based on the use of bevacizumab^39^ (Fig. 1b).

Targeting the VEGF receptor (VEGFR) itself has also been explored^57^. Among the three reported VEGFRs (VEGFR-1, VEGFR-2, VEGFR-3) in mammals, VEGFR-2, whose signaling is triggered by VEGF-A binding, is associated with pathologic angiogenesis^51,58^. Blocking VEGFR-2 activity results in increased oxygenation through vascular normalization in various tumor models. For instance, DC101, a rat monoclonal antibody specific to mouse VEGFR-2, induced vascular normalization not only in GBM but also in lung, breast, and colorectal cancer^46,59^. Moreover, this antibody appeared to enhance drug delivery into tumors in breast and colon carcinomas^60^. Another antibody targeting VEGFR-2 is ramucirumab (Cyramza, ImClone Systems), which shares a similar mechanism with DC101 and may possess the potential for vascular normalization, although limited studies have been conducted on it^61^ (Fig. 1b).

VEGFR-2 functions as a receptor tyrosine kinase (RTK), regulating downstream molecules such as phospholipase γ (PLCγ) and growth factor receptor-bound protein 2 (Grb2)^62^. Several inhibitors have been developed to target the kinase activity of VEGFR-2 and suppress tumor growth. Sunitinib (Sutene, Pfizer), a type I tyrosine kinase inhibitor that binds to the ATP-binding site of the receptor and thereby blocks ATP hydrolysis, is one example. Sunitinib efficiently increases tumor oxygenation in a murine squamous cell cancer model (SCC VII)^63^ and reduces tumor IFP in an orthotopic human glioma model^64^ (Fig.1b). Notably, in the latter model, sunitinib also improves the delivery of the chemotherapeutic drug temozolomide (Temodar, Merck) into the tumor.

### Targeting Ang–Tie signaling restores abnormal tumor vasculature

Another promising target for vascular normalization is the angiopoietin (Ang)–Tie pathway. Among the Ang ligands, Ang2 is often upregulated during tumor angiogenesis. Binding of Ang2 to the Tie2 receptor induces pericyte detachment, vessel regression, and hypoxia^65,66^. Importantly, Ang2 plays a role in the early stages of tumorigenesis, and in Ang2-knockout (KO) mice, the tumor vessel phenotype resembles that of mature blood vessels, indicating that targeting the Ang–Tie pathway can achieve vascular normalization^65,67^.

Concomitant targeting of Ang2 and VEGFR has demonstrated vascular normalization effects in several tumor models^46^. For instance, dual inhibition of VEGFR and Ang2 using cediranib (Recentin, AstraZeneca) in combination with an anti-Ang2-neutralizing antibody or simultaneous targeting of both pathways with a bispecific antibody resulted in an extended normalization window in orthotopic GBM models, leading to prolonged survival^68,69^ (Fig. 1b).

Furthermore, the simultaneous activation of Tie2 and inhibition of Ang2 using an antibody capable of triggering Tie2 phosphorylation through clustering of Ang2 has normalized tumor vessels in orthotopic GBM, subcutaneous Lewis lung carcinoma, and spontaneous mammary cancer models^70,71^. These findings suggest that targeting aberrant blood vessels in tumors by modulating the Ang–Tie pathway and the VEGF pathway may be a promising strategy for restoring the functionality of the tumor vascular bed.

### Targeting notch signaling normalizes tumor vasculature through the action of immune cells

The evolutionarily conserved Notch signaling pathway plays crucial roles in vascular development. Notch signaling begins at four transmembrane receptors (Notch 1–4) activated by five transmembrane ligands (Jagged 1, Jagged 2, Delta-like [DLL] 1, DLL3, DLL4)^23,72^. Among these ligands, DLL1 and DLL4 have been identified as key regulators of tumor angiogenesis, making them promising therapeutic targets^23,73^. However, it is important to note that targeting DLL4 has shown several significant adverse effects^74,75^. Long-term DLL4 blockade can lead to the development of vascular neoplasms, while persistent activation of DLL4 has been associated with T-cell acute lymphoblastic leukemia^23,76,77^.

In contrast, targeting DLL1 has shown promising results. Zhang et al. demonstrated that DLL1 overexpression in E0771 breast cancer models and LAP0297 lung cancer models induced vascular normalization, characterized by a more uniform distribution of functional blood vessels throughout the tumor^23^ (Fig.1b). Additionally, DLL1 overexpression correlated with the normalization of the tumor immune profile, as it polarized tumor-associated macrophages (TAMs) from an immunosuppressive M2-like phenotype to a proinflammatory M1-like phenotype and activated CD8 + T cells. Notably, in vivo depletion of CD8 + T cells or deficiency of interferon-ɣ (IFN-ɣ) nullified the normalization effect observed with DLL1 overexpression, suggesting that CD8 + T cells may be responsible for the normalization of blood vessels and that immune normalization and vascular normalization are interconnected phenomena.

### Oncogenic signaling in cancer cells is an alternative target for vascular normalization

Uncontrolled growth, a hallmark characteristic of cancer, often arises due to genetic alterations in oncogenes, tumor suppressor genes, and stability genes^17,78^. Many signaling pathways, including those upstream of VEGF or other angiogenic factors, are affected by such genetic alterations. One well-known oncogenic signaling pathway that impacts angiogenesis is the epidermal growth factor (EGF)–epidermal growth factor receptor (EGFR)–Ras–phosphoinositide 3-kinase (PI3K)–Akt pathway^79^ (Fig. 1b). Therefore, targeting the components of this pathway can regulate angiogenesis. For example, targeting EGFR with erlotinib (Tarceva, Genentech) induces vascular normalization by reducing the expression of hypoxia-inducible factor-1α (HIF-1α) and VEGF in head and neck squamous cell carcinoma (HNSCC) xenograft models^80^.

Another potential target is H-Ras, which is involved in angiogenesis through the activation of downstream kinases^81^. To become activated and oncogenically transformed, H-Ras requires prenylation by a farnesyltransferase. Inhibiting these posttranslational modifications with farnesyltransferase inhibitors (FTIs) suppresses the growth of Ras-transformed cells^82^. In glioma xenografts, inhibition of Ras prenylation with FTIs has been shown to increase oxygenation, indicating vascular normalization^83,84^.

Indirect inhibition of the Akt pathway has also demonstrated vessel-normalizing effects. The HIV protease inhibitor nelfinavir (Viracept, Pfizer) induces a decrease in VEGF secretion and an increase in oxygenation, similar to the vascular normalization effects observed with bevacizumab^85^.

Qayum et al. demonstrated that vascular normalization can be achieved in HT1080 tumors and spontaneously arising tumors in MMTV-neu mice by targeting EGFR, Ras, PI3K, or Akt^86^. Specifically, they used gefitinib (Iressa, AstraZeneca) to target EGFR, FTI L-778,123 to target Ras, and nelfinavir to target PI3K and Akt. This strategy effectively increased tumor oxygenation, and the normalization window in this study extended from Day 5 after treatment until the end of the study.

The HGF/c-Met pathway, upregulated in various cancers^87–89^, is associated with negative prognostic outcomes^90^. Activation of this pathway promotes cell proliferation and secretion of angiogenic factors, including VEGF ligands. HGF/c-Met signaling synergizes with VEGF signaling to promote angiogenesis and confer resistance against AATs^91–93^. Therefore, targeting the HGF/c-Met pathway has promise for normalizing tumor vasculature^92^. Moreover, HGF’s inhibitory effect on dendritic cell function can lead to the differentiation of immunosuppressive regulatory T cells, again highlighting the interconnectedness between vascular and immune normalization^94^.

### CD4 + T cells can induce vascular normalization

As mentioned above, vascular normalization and immune normalization are closely interconnected concepts. Bioinformatic analyses have revealed correlations between gene expression features associated with vascular normalization and those related to immunostimulatory pathways^95^. Additionally, studies have demonstrated increased vessel permeability and pericyte deficiency in CD4-KO or T-cell receptor (TCR)-KO mice, indicating the crucial roles played by CD4 + T cells in vascular normalization. The adoptive transfer of CD4 + T cells reduces hypoxia in E0771 xenograft models, and immune checkpoint blockade (ICB) induces vascular normalization^95,96^.

Activated CD4 + T-cells colocalize with tumor-associated endothelial cells, causing transcriptomic alterations in these cells. This was confirmed in CD4 + T-cell KO mouse models, which had increased VEGF-A ligand and Ang1/Ang2 ratio, indicating vascular dysfunction^97^. Additionally, the extracellular matrices of tumor-associated cells were disrupted in the absence of CD4 + T cells^95^. Although the specific molecular mechanism is not fully elucidated, these findings make it clear that CD4 + T cells induce vascular normalization via various pathways. Among the diverse CD4 + T cells, Th1 cells and their IFN-γ secretion were the major CD4 + T-cell subtypes associated with vascular normalization. CTLA4 blockade promotes vessel normalization via the accumulation of eosinophils in breast cancer^98^. Additionally, in colorectal cancer, anti-PD-L1 therapy induces tumor vascular normalization via CD8 + T cells, which is antagonized by CD4 + T cells^99^ (Fig. 1b).

### Other strategies that normalize the tumor vasculature

Other than the aforementioned approaches, several strategies have been explored to normalize aberrant tumor vasculature. One such strategy involves the use of nanocarriers that deliver nitric oxide (NO), a molecule known to regulate angiogenesis and maintain vascular homeostasis^100^. Delivery of NO using nanocarriers normalizes tumor vasculature and improves the efficacy of anticancer therapies, particularly in hepatocellular carcinoma^101^. Low-dose NO delivery through this approach can create an immunostimulatory environment, highlighting the intertwined nature of vascular and immune normalization^101^.

Targeting stromal cells, including CAFs and the ECM produced by them, represents another avenue to alleviate solid stress, which can enhance blood perfusion and improve drug delivery^24,102^. Additionally, physical methods such as low-dose gamma radiation or the use of oxygen microbubble delivery with ultrasound have also been explored as vascular normalization strategies^103,104^. Additionally, an emerging strategy for vascular normalization involves the use of microRNAs (miRNAs) that are associated with angiogenesis^105^.

## Normalization of the immune microenvironment

### Background

**Fig. 2:**
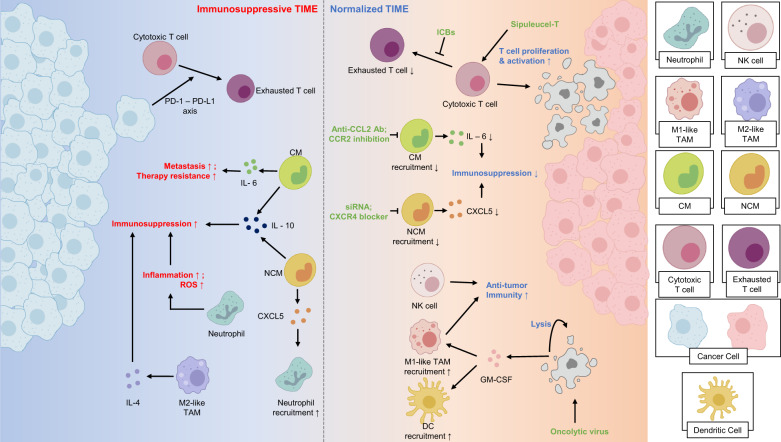
Immune normalization: Modulating the tumor immune microenvironment to suppress immunosuppression and recruit antitumoral immune cells. Tumors employ mechanisms to enhance immunosuppression, including recruiting immunosuppressive leukocytes and suppressing the function of antitumoral immune cells. Chemotaxis of classical monocytes (CMs) is facilitated by the CCL2–CCR2 axis, and these CMs secrete cytokines such as IL-6, that promote tumor metastasis and therapy resistance. Tumors secrete CX3CL1, recruiting nonclassical monocytes (NCMs) that, in turn, can recruit neutrophils via the CXCL5–CXCR2 axis. Both CMs and NCMs secrete immunosuppressive cytokines, including IL-10. M2-like TAMs further suppress the antitumoral immune response by secreting cytokines such as IL-4. Immune checkpoints employed by tumors dampen the function of effector T cells. Strategies for immune normalization include inhibiting monocyte recruitment using anti-CCL2 antibodies/siRNAs, CXCR4 blockers, or CCR2 inhibition. Enhancing effector T-cell function can be achieved through cancer vaccines or immune checkpoint blockade (ICB). Additionally, oncolytic viruses can target tumor cells, inducing their lysis and recruiting antitumoral immune cells. NK, natural killer; CM, classical monocyte; NCM, nonclassical monocyte; TAM, tumor-associated macrophage; ROS, reactive oxygen species; ICB, immune checkpoint blockade; GM-CSF, granulocyte-macrophage colony-stimulating factor.

In many cancer types, tumors mold the TME into an immunosuppressive state characterized by defects in antigen presentation machinery and the recruitment of immunosuppressive cells. These features are often observed in the TME of solid tumors and are influenced by factors such as hypoxia and high interstitial fluid pressure (IFP)^106^. For example, hypoxia leads to increased activation of hypoxia-inducible factor-1 (HIF-1) and vascular endothelial growth factor-A (VEGF-A), which in turn result in the expression of inhibitory checkpoints in CD8 + T cells, reduced functionality of dendritic cells, and the recruitment and polarization of myeloid cells into immunosuppressive phenotypes^25,107–109^. The high IFP in solid tumors stimulates CAFs to produce transforming growth factor-beta (TGF-β), which promotes the differentiation and proliferation of regulatory T helper cells (Tregs), induces the generation of protumorigenic myeloid cell phenotypes, and accelerates desmoplasia^2,110^ (Fig. 2).

Normalization of the immune cell profile within the TME is defined differently by different research groups. Sanmamed et al. describe normalization in the context of immunotherapy, categorizing strategies into “enhancement therapies” that activate a general systemic immune response and “normalization therapies” that target specific dysfunctional immune responses^111^. In this review, we will use a broader definition, considering the transition of the TME from an immunosuppressive microenvironment to a tumor-suppressing phenotype as normalization (Fig. 2).

Given the promising results of immunotherapy against various cancers, several anticancer therapies harnessing the immune system have been developed. Therefore, we will explore some of these methods, including immune checkpoint blockade, reprogramming of myeloid cell populations, and cancer vaccines.

### Immune checkpoint blockade restores the function of antitumoral immune cells

CD8 + T cells are immune cells that play a crucial role in exerting antitumoral effects, primarily by eliminating tumor cells through mechanisms such as perforin/granzyme secretion or FasL/TNF-related apoptosis-inducing ligand (TRAIL)-mediated apoptosis^2^. Upon activation, CD8 + T cells also upregulate immune checkpoint molecules, including cytotoxic T lymphocyte antigen-4 (CTLA-4)^112^. CTLA-4 binds to the same ligands as B7 costimulatory receptors with higher affinity, resulting in reduced T-cell proliferation and diminished secretion of inflammatory cytokines^113^. Another important immune checkpoint molecule is programmed death-1 (PD-1), which is expressed on activated T cells. PD-1 binds to its ligands PD-L1 or PD-L2, which are expressed on various cells within the TME, leading to T-cell inhibition^114^. Current immunotherapies targeting these “exhaustion” mechanisms by blocking CTLA-4 or PD-1 signaling can reverse the exhaustion of effector T cells and enhance their antitumor activity^115^ (Fig. 2).

Several other immune checkpoint molecules have been identified, and some of them are being evaluated as potential therapeutic targets. One such molecule is lymphocyte activation gene-3 (LAG-3), which is expressed on Tregs. LAG-3^high^ Tregs secrete immunosuppressive cytokines, and blockade of LAG-3 signaling using an anti-LAG-3 antibody slows tumor growth. The blockade of immune checkpoint molecules represents a promising strategy in immunotherapy, aiming to unleash the full potential of the immune system against tumors.

### Myeloid cells are key targets in normalizing the immune environment

The myeloid cell population within the TME is highly heterogeneous and has a role in modulating tumor progression^116–118^. However, the significance of myeloid cells in immunotherapy is often underestimated, and current approaches targeting these cells have shown limited effectiveness^119^.

Monocytes are myeloid cells that can be classified into two subtypes, classical and nonclassical^120^. Classical monocytes (CD14 + CD16- in humans, Ly6C^hi^ CCR2+ in mice) have been generally associated with protumoral functions, including promoting cancer metastasis and increasing therapy resistance^121,122^. Strategies targeting the recruitment of classical monocytes in mice through CCR2 inhibition or the use of anti-CCL2 antibodies have shown promise in creating an antitumoral TME and reducing therapeutic resistance, respectively^122,123^. One limitation of this approach is that termination of anti-CCL2 treatment in certain tumors has led to accelerated tumor metastasis and worse outcomes due to increased IL-6 within the tumors^124^ (Fig. 2).

Nonclassical monocytes, defined as CD14- CD16+ in humans and Ly6C^lo^ CX3CR1+ in mice, can enhance resistance to anticancer therapy. AATs can induce tumors to secrete CX3CL1, which increases the recruitment of nonclassical monocytes. These monocytes can enhance the infiltration of neutrophils into the TME through the CXCL5–CXCR2 axis^117,118^. Both nonclassical monocytes and neutrophils exert protumoral functions by secreting immunosuppressive cytokines such as IL-10. Several methods targeting these cells, such as the use of siRNAs or CXCR4 blockers, have shown promising results^118^ (Fig. 2).

The function of monocytes in the TME is context-dependent, and they have also been reported to play antitumoral roles in certain circumstances. For example, infiltration of classical monocytes into KPC tumors, a murine pancreatic ductal adenocarcinoma (PDAC) model, has led to a reduction in tumor fibrosis, increasing the efficacy of chemotherapy^125^. Nonclassical monocytes can inhibit metastasis and activate natural killer (NK) cells, contributing to cancer immunosurveillance^126,127^ (Fig. 2).

Neutrophils are another myeloid cell population that can exert protumoral functions within the TME. Recruited neutrophils can promote tumorigenesis by facilitating inflammation or releasing reactive oxygen species. They promote tumor proliferation, angiogenesis, and metastasis through diverse mechanisms^120^ (Fig. 2). Therefore, reducing the neutrophil population or inhibiting their function within the TME may help create an immune environment favorable for anticancer treatments. In HER2-negative gastric cancers, which exhibit a limited response to current therapies, increased infiltration and protumoral activities of neutrophils and nonclassical monocytes have been observed, highlighting the potential of targeting these cell types as novel therapeutic approaches^128^. Emerging evidence has demonstrated the significant role of neutrophil extracellular traps (NETs) in promoting tumor metastasis, further underscoring the criticality of targeting myeloid cells within the TME^129,130^. To fully exploit the therapeutic potential of the monocyte and neutrophil populations within the TME, a comprehensive understanding of their context-dependent functions is needed, and detailed reviews of their important properties have been provided by Jeong et al.^120^.

Macrophages are another myeloid cell population that deserves attention. High infiltration of macrophages has been associated with a negative cancer prognosis^131^. TAMs are highly heterogeneous and can be divided into two subtypes, although these definitions are oversimplified: inflammatory M1-like TAMs and immunosuppressive M2-like TAMs. The TME promotes the polarization of TAMs toward a more M2-like phenotype through hypoxia or signaling by immunosuppressive cytokines, including IL-4^132^. This makes TAMs attractive TME targets. For example, the chemokine CCL2 recruits CCR2+ monocytes, which differentiate into macrophages, and blocking CCR2 has prevented immune suppression in some preclinical models, improving chemotherapeutic efficacy^133^. Since M2-like TAMs, not M1-like TAMs, drive immune suppression, inhibiting polarization toward an M2-like phenotype is another potential mechanism for successful treatment. Targeting the metabolic pathways or essential transcription factors of TAMs has demonstrated antitumoral effects in various mouse models^134^ (Fig. 2).

Myeloid cells can transform into malignant cells, leading to acute myeloid leukemia (AML) or chronic myeloid leukemia (CML)^135,136^. AML is characterized by the infiltration of malignant hematopoietic cells into various tissues, including bone marrow and blood^137^. While conventional chemotherapy is commonly used for AML, emerging research suggests that immunotherapies targeting surface molecules, chimeric antigen receptor (CAR) T-cell therapy, and cancer vaccines could be strategies for AML treatment^138^. CML is a slower-developing blood cancer similar to AML^139^. Studies investigating the effects of immunotherapy for CML have explored immunostimulatory treatments such as tyrosine kinase inhibitors, IFN-γ therapy, and ICBs^140^. Since this review focuses on solid cancers, we will not delve further into blood cancers.

### Recruitment of antitumoral immune cells normalizes the protumoral immune profile

Modulating the immune profile of the TME by recruiting antitumoral immune cells can create a favorable environment for cancer treatment. One approach to achieve this is through the use of oncolytic viruses (OVs). OVs are either genetically modified or naturally occurring viruses that selectively replicate in cancer cells to kill the tumor without harming normal cells^141^. Talimogene laherparepvec (T-VEC, Imlygic) is a recombinant herpes simplex virus (HSV)-1 engineered to express human granulocyte–macrophage colony-stimulating factor (GM-CSF). It replicates within tumor cells, causing their lysis and the release of GM-CSF. This recruitment of GM-CSF promotes the maturation of dendritic cells (DCs) and macrophages, establishing a robust antitumor immune response^142^ (Fig. 2).

Inflammatory cytokines can also be utilized to recruit antitumoral immune cells. Interferon-α (IFN-α), one of the first cytokines approved for cancer therapy, upregulates MHC class I expression, induces caspase-dependent apoptosis, and attenuates tumor angiogenesis^143^. Although IFN-α treatment has shown effectiveness as a single agent, its effects are enhanced when combined with other anticancer drugs^144^. Another cytokine, interleukin-2 (IL-2), can recruit antitumoral immune cells. High-dose IL-2 treatment has demonstrated promising results in metastatic renal cell carcinoma and melanoma^145,146^.

Physical methods can be employed to recruit immune cells. For example, mild photothermal therapy has been shown to effectively increase the infiltration of lymphocytes into tumors and enhance antitumoral T-cell activity^147^.

### Adoptive transfer of engineered immune cells is a promising normalization method in blood cancers

Forming an antitumoral immune environment can also be achieved through the adoptive transfer of autologous immune cells that have been isolated, engineered, and expanded in advance to generate durable antitumor immune responses^148^. One example of this approach is CAR T-cell therapy, which involves transferring T cells engineered with synthetic receptors capable of recognizing tumor-associated antigens (TAAs) to kill tumor cells^149^. CAR T cells utilize cytotoxic pathways similar to those employed by effector CD8 + T cells, including the secretion of perforin and granzymes and the activation of apoptotic pathways through death receptors^150^. CAR T-cell therapy targeting the CD19 molecule on B cells has demonstrated effectiveness in eradicating lymphoma^151^.

Although CAR T-cell therapy has shown promising results, it also has limitations. In many solid tumors, CAR T-cell therapy exhibits low efficacy due to limited infiltration^152^. However, considering the overall advantages and effectiveness of CAR T-cell therapy, efforts to improve its performance in solid tumors have been pursued through various approaches, such as expressing CCR2 or overexpressing heparanase (HPSE)^153,154^.

### Cancer vaccines boost the function of antitumoral immune cells

Cancer vaccines are designed to enhance antitumor immune responses to tumor-associated antigens (TAAs) by modulating the antigen-presenting function of antigen-presenting cells (APCs) and establishing durable antitumor memory^155^. Sipuleucel-T (Provenge, Dendreon) is an FDA-approved therapeutic cancer vaccine used for prostate cancer. It is produced by culturing autologous peripheral-blood mononuclear cells (PBMCs) and activating them with engineered antigens containing the TAA prostatic acid phosphatase (PAP)^156^. Treatment with Sipuleucel-T prior to prostate cancer surgery has been shown to enhance T-cell proliferation, activation, and infiltration.

Cancer vaccines are expected to synergize with immune checkpoint inhibitors (ICBs) since their ultimate goal is to enhance adaptive immunity^157^. Additionally, cancer vaccines have the potential to induce long-lasting antitumor efficacy by triggering immunological memory. The efficacy of cancer vaccines can be compromised by various factors within the TME, such as the local accumulation of immunosuppressive myeloid cells and Tregs, as well as downregulation of antigen expression and alterations in the antigen processing pathway^155^.

### The immunogenic cell death and STING pathways are promising targets for immune normalization

Another approach to normalize the immune microenvironment is through controllable lytic cell death, which induces immunogenic cell death (ICD)^158^. Controllable ICD pathways, such as necroptosis, ferroptosis, and pyroptosis, trigger the release or surface expression of damage-associated molecular patterns (DAMPs), which can function as adjuvants or danger signals to enhance the immune response against tumors^159^. Several anticancer agents currently used in the clinic, including such chemotherapeutic agents as oxaliplatin, induce hallmarks of ICD, suggesting their potential as vaccines for inducing protective anticancer immune responses^160,161^.

A second method of immune normalization is targeting the STING pathway, which plays a crucial role in the antitumoral response of CD8 + T cells in several cancer models^162^. Notably, the expression of STING in endothelial cells has been correlated with better prognosis in human colon and breast cancer^163^. Targeting the STING pathway not only helps normalize the immune environment but also promotes vascular normalization. Specifically, intratumoral STING activation normalizes the tumor vasculature through type I IFN signaling and CD8 + T cell activity^163^.

## Conclusions

Vascular normalization holds promise as an explanation for the synergistic effects observed when combining AATs with chemotherapy and immunotherapy, which are not as effective when used alone. There is still debate about the actual benefits of this strategy. For instance, in cerebral tumors, vascular normalization may restore the low permeability of the brain vasculature, potentially hindering the delivery of therapeutic agents and reducing the effectiveness of other treatments^164^. Similarly, vascular normalization with bevacizumab in xenograft models of ovarian and esophageal cancer leads to decreased uptake of antibodies, highlighting the need for careful consideration when combining AATs with other therapies^165^. Nonetheless, there is ongoing discussion about whether the dose of bevacizumab used in that trial was too high or appropriate. These debates underscore the necessity for clear guidelines that recommend an effective dose, timing, and duration of treatment that will enhance blood vessel function while minimizing adverse effects.

Another challenge is the concept of the normalization window, which varies significantly between patients and poses a significant obstacle to clinical application. Therefore, the development of strategies aimed at improving blood vessel function, rather than relying solely on existing drugs, may be necessary.

In relation to immune normalization, the heterogeneity of immune cell populations in the TME plays a crucial role in the response to anticancer therapies^166,167^ (Fig. 2). Recent advancements in single-cell omics technologies have provided insights into this diversity^168^, but our understanding is far from complete. Moreover, even cell types that were previously thought to be well defined are composed of heterogeneous subsets. For example, the current classifications of M1-like and M2-like TAMs and myeloid-derived suppressor cells (MDSCs) may oversimplify the sophisticated phenotypes/functions of these cells within tumors. Therefore, to better normalize the TIME, a deeper understanding of the functions of diverse immune cell subtypes is needed.

Many immune cell types exhibit context-dependent functions in tumors, meaning that the same subtype may have different effects depending on the tumor type. Inflammatory immune cells, for instance, are often considered beneficial cells that target tumor cells. However, in PDAC, the well-known inflammation has been shown to enhance desmoplasia and accelerate cancer cell proliferation^169^. Therefore, considering the diverse roles of immune cells in various cancer types is crucial for harnessing the immense potential of immune cells within the TME to combat the tumor.

As mentioned above, vascular normalization and immune normalization are intertwined strategies, each strategy impacting the other. Vascular normalization improves vessel function, thereby influencing the immune cell population in the TME. Conversely, immune cells can also induce vascular normalization. Based on these findings, several clinical trials evaluating the safety and efficacy of AATs in combination with immunotherapies have been conducted in various tumor types (Table 1). Targeting CAFs not only affects vascular normalization but also impacts immune cells, potentially enhancing the effects of immunotherapies. Some reports suggest that the effects of CAF-targeting antibodies are mediated by CD8 + T cells, further highlighting the close relationship between vascular and immune normalization^170^.

**Table 1:** List of clinical trials combining antiangiogenic therapies and immunotherapies.

Tumor Type	Clinical Trial ID	AAT	IT	Other therapies included in the combination^a^
Malignant Solid Tumor	NCT02857920	Bevacizumab	Allogenic NK therapy	No
Hepatocellular carcinoma/Bilary tract cancer	NCT04518852	Sorafenib	PD-1 mAb^b^	Yes
	NCT04273100	Lenvatinib	PD-1 mAb^b^	Yes
	NCT05313282	Apatinib	Camrelizumab	Yes
	NCT05775159	Bevacizumab Lenvatinib	MEDI5752	Yes
	NCT05665348	Bevacizumab	Atezolizumab Ipilimumab	No
	NCT05750030	Bevacizumab	Atezolizumab	Yes
	NCT04191889	Apatinib	Camrelizumab	Yes
	NCT05052099	Bevacizumab	Atezolizumab RP3	No
	NCT05733598	Bevacizumab	Atezolizumab	Yes
	NCT03937830	Bevacizumab	Durvalumab Tremelimumab	Yes
	NCT02519348	Bevacizumab	Durvalumab Tremelimumab	Yes
	NCT05359861	Bevacizumab	Atezolizumab SRF388	No
Ovarian cancer	NCT04928583	Bevacizumab	Oregovomab	Yes
	NCT02759588	Bevacizumab	GL-ONC1	No
	NCT03197584	Bevacizumab	Avelumab	Yes
	NCT05281471	Bevacizumab	Olvimulogene nanivacirepvec	Yes
Gastrointestinal cancer	NCT04069273	Ramucirumab	Pembrolizumab	Yes
Lung cancer	NCT05360979	Recombinant human endostatin	Envafolimab	Yes
	NCT05529355	Endostar	Envafolimab	Yes
	NCT04459078	Apatinib	Camrelizumab	Yes
	NCT03280563	Bevacizumab	Atezolizumab	Yes
	NCT05781308	Bevacizumab	Atezolizumab	No
	NCT05782764	Endostar	ICB^b^	Yes
	NCT04303130	Endostar	Camrelizumab	No
	NCT03169738	Bevacizumab	Avelumab	Yes
	NCT02039674	Bevacizumab	Pembrolizumab	Yes
Pancreatic cancer	NCT05298020	Endostar	Envafolimab	Yes
	NCT03136406	Bevacizumab	Several^c^	Yes
	NCT03329248	Bevacizumab	Avelumab	Yes
	NCT03387098	Bevacizumab	Several	Yes
	NCT03193190	Bevacizumab	Several	Yes
Breast cancer	NCT04877821	Anlotinib	Sintilimab	No
	NCT03395899	Bevacizumab	Atezolizumab	Yes
	NCT0518006	Bevacizumab	Atezolizumab	No
	NCT03175666	Bevacizumab	Avelumab	Yes
	NCT04739670	Bevacizumab	Atezolizumab	Yes
	NCT03387085	Bevacizumab	Several^c^	Yes
Colorectal cancer	NCT03169777	Bevacizumab	Several^c^	Yes
	NCT04262687	Bevacizumab	Pembrolizumab	Yes
	NCT03950154	Bevacizumab	PD-1-T cell	Yes
	NCT04017455	Bevacizumab	Atezolizumab	No
	NCT05733611	Bevacizumab	Atezolizumab	Yes
	NCT04527068	Bevacizumab	Tripleitriumab	No
	NCT04194359	Bevacizumab	Sintilimab	No
Blood cancer	NCT03169790	Bevacizumab	Avelumab	Yes
	NCT03167177	Bevacizumab	Avelumab	Yes
Nasopharyngeal cancer	NCT03813394	Bevacizumab	Pembrolizumab	No
GBM	NCT04952571	Bevacizumab	Camrelizumab	No
Skin cancer	NCT03167164	Bevacizumab	Avelumab	Yes
	NCT03387111	Bevacizumab	Several	Yes
Urothelial cancer	NCT03197571	Bevacizumab	Avelumab	Yes
Head and Neck Squamous Cell Carcinoma	NCT03193190	Bevacizumab	Avelumab	Yes

*AAT* anti-angiogenic therapies; *IT* immunotherapy.

Clinical trials are included if the trial title mentions a combination of AAT and IT. *Resource:* http://clinicaltrials.gov.

^a^Other therapies include chemotherapy and transarterial chemoembolization.

^b^Specific therapeutics used are not clarified.

^c^More than two types of immunotherapies were utilized.

Therefore, instead of pursuing a single strategy, targeting both vascular and immune normalization simultaneously may yield even greater efficacy. Studies evaluating the effects of different combinations of strategies are warranted to fully explore the potential benefits of this approach.
